# Recent advances in isothermal amplification techniques for detection of animal diseases

**DOI:** 10.3389/fvets.2026.1819190

**Published:** 2026-05-07

**Authors:** Fahimeh Tooryan, Ben Enyertornye, Farhatul J. Choudhury, Binu T. Velayudhan

**Affiliations:** Athens Veterinary Diagnostic Laboratory, College of Veterinary Medicine, University of Georgia, Athens, GA, United States

**Keywords:** animal pathogens, diagnostics, food animal, isothermal amplification, point-of-care testing

## Abstract

Prompt pathogen detection is critical in protecting public health, improving animal health, welfare, food safety, and food security in both food-producing and companion animals. Although conventional and molecular diagnostic methods are highly effective, they often require expensive instrumentation, specialized laboratory infrastructure, and trained personnel, limiting their applicability in field and resource-limited settings. Isothermal amplification technologies have emerged in recent times as powerful alternatives to conventional PCR-based methods, offering rapid, sensitive and user-friendly nucleic acid detection without the need for thermocycling equipment. The present review summarizes recent advances in isothermal amplification methods and their applications in the detection of pathogens in livestock and companion animals. It also highlights innovations in assay design, improvements in analytics, and their incorporation into portable and field-deployable detection platforms. Furthermore, the review explores the challenges and future directions for the application of these technologies in routine veterinary diagnostics and integrated disease surveillance programs.

## 1 Introduction

Livestock diseases have a major negative impact on global food security, public health and economic stability (1). Meanwhile, these animals are faced with diseases which can be due to nutritional deficiencies, metabolic and endocrine issues, genetic defects, environmental and husbandry-related issues, or infectious in nature. Infectious diseases in livestock can lead to severe consequences, including reduced productivity, increased mortality, substantial financial losses, animal suffering and welfare concerns, public health and zoonotic risks, and erosion of trust in veterinary infrastructure. These impacts extend beyond individual farms to the point of influencing trade, market prices, and food availability. Early detection and containment of outbreaks are therefore essential, as timely intervention can halt disease transmission dynamics and significantly reduce its negative impacts on a larger scale (2).

The economic burden of animal diseases underscores the urgency for rapid diagnostics. For instance, paratuberculosis (Johne’s disease) herds experience an average loss of approximately US$100 per cow, while high-prevalence herds (≥10% clinical culls) suffer losses exceeding US$200 per cow. Across the U. S. dairy industry, the average annual loss is estimated to be US$250 million annually (3). On a global scale, the combined economic cost of *Mycobacterium tuberculosis* and mastitis to the cattle industry is approximately US$35 billion (4, 5). In China, the economic loss due to African swine fever in 2019 was estimated to be US$111.2 billion (6). These staggering figures highlight the need for diagnostic tools that enable early detection and rapid response to prevent widespread economic and health consequences.

Rapid diagnostics are not only vital for food-producing animals but also for companion animals. In pets, timely pathogen detection enhances tailored veterinary care, improves treatment outcomes, and supports the One Health concept by facilitating surveillance of zoonotic diseases. Portable and cost-effective diagnostic tools are transforming global disease surveillance, improving animal welfare, and strengthening food security.

Despite the importance of rapid diagnostics, conventional polymerase chain reaction (PCR) remains the gold standard for pathogen detection in many veterinary establishments. However, PCR-based methods have notable limitations that hinder their widespread use in field settings. These include the requirement for expensive thermal cyclers and laboratory infrastructure, reliance on skilled personnel for sample processing and interpretation, and time-consuming protocols that delay decision-making (7). Additionally, clinical specimens often carry a variety of substances that can inhibit PCR amplification, including heme, urea, heparin, immunoglobulin G, lactoferrin, and various polysaccharides (8). Also, non-specific annealing of primers and increased risk of false positives result further hinders the development of PCR-based methods for field diagnostics (9). Such constraints make PCR impractical for on-site testing, particularly in resource-limited environments or during emergency outbreaks. Meanwhile, there are assertions that isothermal amplification assays (such as LAMP) are more robust to inhibitors which are commonly present in clinical samples in comparison to PCR. This provides another justification for their use in screening under field conditions (10).

To overcome these challenges presented by conventional PCR, emerging isothermal amplification strategies have gained attention as promising alternatives for rapid pathogen detection. Unlike PCR, these methods operate at a constant temperature, eliminating the need for complex thermal cycling equipment. Methods such as loop-mediated isothermal amplification (LAMP), recombinase polymerase amplification (RPA), and nucleic acid sequence-based amplification (NASBA) offer rapid turnaround times, high sensitivity, and compatibility with portable devices. For instance, recent studies have demonstrated that RT-RPA assays provide a simple, rapid, and reliable method for detecting canine distemper virus (CDV) both in laboratories and point-of-care facilities, particularly in resource-limited settings. Such tools could be invaluable at points of need, such as animal shelters, where rapid isolation and outbreak control are critical (11).

In addition to isothermal amplification, several advanced NA detection technologies are widely used in veterinary diagnostics, including quantitative real-time PCR (qPCR), digital PCR (dPCR), and fluorescent probe-based assays. As core tools in modern molecular diagnostics, these methods differ in sensitivity, specificity, quantification capability, and operational complexity.

qPCR is widely regarded as the gold standard due to its high sensitivity, specificity, and real-time quantitative capability through fluorescence-based monitoring (12). However, its dependence on precise thermal cycling, sophisticated instrumentation, and trained personnel limits its use in decentralized and resource-limited settings (13). dPCR enables absolute quantification with enhanced sensitivity by partitioning samples into numerous microreactions, making it particularly effective for detecting low-abundance targets (14). Nevertheless, high cost and technical complexity restrict its broader application.

Fluorescent probe-based systems, such as TaqMan and molecular beacons, further improve specificity but remain reliant on advanced laboratory infrastructure (13). In contrast, isothermal amplification methods represent a transformative alternative by eliminating the need for thermal cycling and operating under constant temperature conditions. These methods significantly reduce equipment requirements, simplify workflows, and enable rapid turnaround times. Importantly, their adaptability to point-of-care (POC) platforms and resource-limited environments positions them as highly accessible and scalable diagnostic solutions, with growing potential to complement or even replace conventional PCR-based approaches in field and decentralized settings (15).

Overall, while PCR-based methods remain the benchmark for analytical accuracy and standardization, isothermal technologies uniquely address critical gaps in accessibility, speed, and field applicability, underscoring their increasing importance in next-generation diagnostic strategies.

Isothermal amplification technologies offer significant advantages in terms of cost, portability, and on-site applicability. Unlike conventional PCR, these methods are performed at constant temperatures and do not require expensive thermocyclers, thereby enabling the use of simple thermal devices and reducing overall cost (16). Methods such as LAMP and RPA allow rapid detection with minimal infrastructure requirements and are well-suited for decentralized and resource-poor settings (17). Furthermore, the high stability and tolerance of these methods to inhibitors present in the sample allow for direct testing with minimal sample preparation, enhancing their applicability in field and point-of-care settings (18). Furthermore, the recent integration of these technologies with CRISPR systems and microfluidic platforms has increased portability, sensitivity, and ease of use, further enhancing their potential for rapid on-site detection of pathogens (19).

In this review, we explore the principles, advantages, challenges, and applications of emerging isothermal amplification strategies in veterinary diagnostics. Highlighting the transformative potential of these technologies, we aim to emphasize their role in improving disease surveillance, animal health, and global food security.

## 2 Overview of isothermal amplification technologies

Isothermal amplification of nucleic acids (NA) refers to a set of advanced biomolecular methods that amplify DNA and/or RNA at a constant temperature, eliminating the need for the sequential thermal cycles of denaturation, primer annealing, and extension common to the polymerase chain reaction (PCR). In these systems, the amplification process is carried out continuously under uniform thermal conditions, thereby increasing the reaction speed and efficiency. These technologies are based on enzymatic mechanisms of double-stranded unwinding or strand exchange and utilize strand-displacing DNA polymerases. These enzymes allow continuous unwinding of double-stranded templates and continuous primer extension without temperature changes, resulting in exponential amplification of NA under thermally stable conditions. Isothermal amplification reactions are typically performed at approximately 25–70 °C, depending on the platform and enzyme used. This feature simplifies the equipment required, reduces operational complexity, and facilitates the development of rapid, low-cost, and portable diagnostic systems for laboratory and field applications (20–22). Recent reviews have classified isothermal NA amplification methods into distinct functional categories based on the molecular mechanism of single-stranded template generation and the manner in which the amplification process is maintained and sustained (22, 23).

## 3 Mechanism-based classification of isothermal amplification methods

### 3.1 Strand-displacement polymerase systems

Strand-displacing polymerases are enzymes that can build a new DNA strand while pushing aside the existing complementary strand, so there is no need for the high-temperature denaturation step used in PCR. As a result, amplification can occur continuously at a constant temperature. Enzymes such as Bst and Φ29 DNA polymerases are especially effective in this role due to their strong strand-displacement ability, high efficiency, and stability. These features make them essential for isothermal methods such as LAMP, RCA, and MDA, enabling rapid amplification with simpler equipment and making these methods well-suited for field and point-of-care applications (16, 24). This group includes methods such as loop-mediated isothermal amplification (LAMP), rolling circle amplification (RCA), multiple displacement amplification (MDA), and cross-priming and strand exchange (CPA/SEA). These systems use polymerases such as Bst and Φ29 DNA polymerases that can physically displace downstream strands as a new strand is synthesized. This feature allows the amplification process to proceed continuously and at high speed at a constant temperature, resulting in rapid, often exponential, accumulation of double-stranded products (21, 25, 26).

### 3.2 Helicase-based systems

In helicase-dependent amplification (HDA), the DNA helicase is used in conjunction with single-stranded DNA-binding proteins (SSBs) and a polymerase to unwind the two DNA strands, in a manner similar to natural replication in the cell. The continuous activity of the helicase creates single-stranded regions in the DNA template that allow primers to bind and then be extended by the polymerase. This process results in exponential DNA replication at temperatures ranging from about 37 to 65 °C (21, 27, 28).

### 3.3 Recombinant-based systems

In recombinant polymerase-mediated amplification (RPA) and similar systems, recombinant proteins bind primers and form nucleoprotein structures that search for homologous sequences in double-stranded DNA. After recognizing the target sequence, the primers penetrate the double-stranded structure and displace the original strand. A polymerase with strand-displacing capability then extends to the primers. These reactions are typically performed at 37 to 42 °C and allow for very rapid NA amplification, often within 10 to 20 min (21, 29). In addition to RPA, several closely related recombinase-based amplification methods have been developed, with recombinase-aided amplification (RAA) emerging as one of the most widely used, particularly in veterinary diagnostics. RAA operates through a mechanism similar to RPA, in which recombinase proteins facilitate primer targeting and invasion into double-stranded DNA, enabling amplification via strand displacement under isothermal conditions (typically 37–42 °C). This process is rapid and efficient, often producing results within 15–30 min without the need for complex laboratory equipment. Owing to its simplicity and speed, RAA has been increasingly used to detect important animal pathogens such as Newcastle disease virus and avian influenza virus, demonstrating strong performance in field settings (29–31). In addition, integration of RAA with lateral flow assays and portable detection platforms has further enhanced its usability, making it a practical tool for point-of-care diagnostics and on-site disease surveillance in animal health.

### 3.4 RNA transcription-based systems

Nucleic acid sequence-based amplification (NASBA) and similar transcription-mediated systems have been specifically designed to identify RNA targets. These systems utilize a combination of reverse transcriptase, RNase H, and T7-dependent RNA polymerase to generate numerous new RNA copies from a starting RNA template at a constant temperature (approximately 41 °C). Although NASBA was first introduced many years ago, its principles of operation and diagnostic applications have been comprehensively reviewed and summarized in recent reviews (21, 22). Because isothermal NA amplification methods require only a fixed incubation temperature, they can be implemented using simple equipment such as thermal blocks, low-cost dry baths, microfluidic chips, lateral-flow strip-based systems, or integrated portable devices. This feature significantly facilitates the deployment and exploitation of these technologies in decentralized and resource-limited environments, such as farms, slaughterhouses, and small veterinary clinics (25, 26, 30).

**Figure 1:**
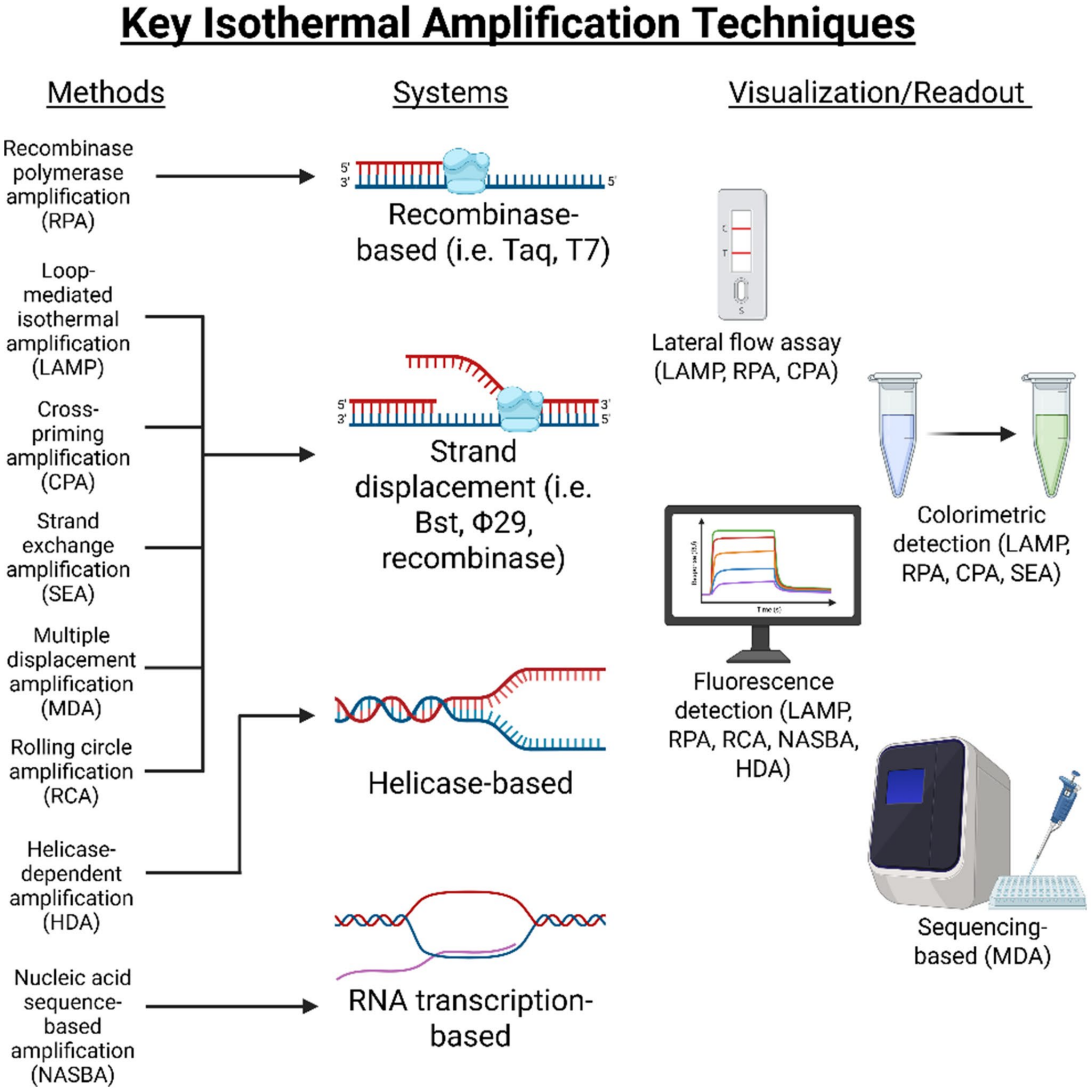
Overview of main isothermal amplification methods, categorized by polymerase activity and corresponding visualization and readout strategies. Created in BioRender. Choudhury (2026) https://BioRender.com/k2zkgpj.

In veterinary diagnostics and food safety, this operational simplicity is a key factor in the widespread adoption of this technology. Methods such as LAMP, RPA, RCA, and NASBA have been successfully used to identify pathogens and determine animal species directly in samples such as meat, milk, feces, and environmental samples, allowing rapid and reliable results with minimal equipment and short turnaround times (25, 26, 31). In all of these platforms, key enzymes include strand-displacing DNA polymerases such as Bst and Φ29, which can remove downstream strands as new DNA is synthesized. In addition, accessory proteins that are responsible for strand separation and stabilization also play a crucial role, including helicases in the HDA system, recombinase and single-stranded DNA binding (SSB) proteins in the RPA system, and multicomponent transcription enzyme complexes in the NASBA method (22, 23, 32). The combination of these mechanical and enzymatic innovations explains why isothermal amplification of NA is now recognized as one of the pivotal platforms in the development of modern diagnostic systems for point-of-care and field applications, which play an important role in monitoring the health of livestock and pets. Figure 1 depicts the mechanistic classification of the major isothermal amplification methods based on polymerase activity, along with the corresponding methods for detection and result reading.

## 4 Key isothermal amplification techniques

### 4.1 Loop-mediated isothermal amplification (LAMP)

LAMP is a technique that uses four to six specific primers to recognize six to eight distinct regions of the target sequence. These primers, together with a strand-transfer-capable polymerase, usually Bst DNA polymerase, form self-priming stem-loop structures and enable rapid, exponential NA amplification under isothermal conditions at approximately 60–65 °C. The reaction products can be detected by techniques such as turbidimetry, DNA-binding fluorescent dyes, or colorimetric markers. In LAMP-based methods, the interpretation of results is inextricably linked to the type of detection system used and can be visual or signal-based. Depending on the assay design, outputs can be assessed by colorimetric changes, real-time fluorescence signals, lateral flow assays, or by the increase in turbidity resulting from the formation of amplification products. This variety of detection formats allows for the selection of rapid, simple, and reliable methods that, in many cases, do not rely on sophisticated laboratory equipment. As a result, LAMP has emerged as a flexible diagnostic platform for point-of-care applications and field monitoring, especially in resource-limited settings (33–37). The RT-LAMP version also allows for direct detection of RNA targets (18, 38). Due to its short response time (30–45 min), relative tolerance to inhibitors in raw samples, and the ability to perform the reaction with simple thermal equipment, the LAMP technique has been widely used for the detection of animal and foodborne pathogens. Recent reviews have reported the development of multiplex assays, contamination control solutions, and one-pot reaction formats for field applications and point-of-care diagnostics (18, 38). The most important advantages of the LAMP technique include its high amplification speed, compatibility with raw samples, and the ability to use a variety of visual detection systems. However, the complex design of primers and the risk of non-specific amplification, including primer-dimer formation and cross-contamination if closed systems are not used, are limitations of this technique (18).

### 4.2 Recombinase polymerase amplification (RPA)

RPA is an advanced isothermal amplification system for NA that combines the natural strand-invasion mechanism with DNA synthesis by polymerases capable of strand displacement. In this system, recombinant proteins first bind to primers and form nucleoprotein complexes that are able to search for homologous sequences in double-stranded DNA. After recognizing the target sequence, these primers penetrate into the double-stranded structure and displace the complementary strand. Single-stranded DNA-binding proteins (SSBs) then stabilize the displaced strand, and a polymerase capable of strand displacement extends the primers and initiates replication (39). The RPA reaction is usually performed at low temperatures of about 37 to 42 °C and can produce detectable amounts of amplification product in as little as 10 to 20 min. The RT-RPA format also allows direct detection of RNA targets by adding a reverse transcription step (39).

Due to the low temperature requirement, very short response time, and the ability to perform the reaction with simple thermal equipment, the RPA technique has become a very suitable option for decentralized diagnostics in environments such as livestock farms, slaughterhouses, and field veterinary centers. This platform has been widely developed for the detection of veterinary pathogens and foodborne pathogens and is often combined with lateral flow strip-based detection systems to allow direct observation of results without the need for laboratory equipment. Comprehensive reviews published between 2017 and 2022, as well as updates in 2024, have summarized advances in assay design, reaction kinetics, inhibitor tolerance, and the development of multiplex assays (40–42).

The ability to perform the reaction at near-ambient temperatures allows RPA to be performed with very simple heat sources, including small thermal blocks or even body-heat-based systems. This feature makes RPA a very attractive option for point-of-care veterinary diagnostics. The rapidity of result acquisition also makes this technique an ideal tool for field screening of livestock pathogens and companion animal infections. However, the relatively high cost of enzymes and the need for careful primer and probe design to reduce non-specific amplification are among the technical challenges of this system (40–43). The most important advantages of the RPA technique include its very low operating temperature, extremely high speed, and the ability to perform the reaction with simple equipment. On the other hand, achieving high analytical specificity requires careful optimization of primers and probes, and patent and commercial licensing considerations may affect industrial development and widespread application of this technology (40–42). Amplification products from RPA technology are mainly detected by fluorescence-based detection systems or lateral flow assays, which allow for rapid, accurate, and user-friendly interpretation of results in field conditions and point-of-care environments. By providing visual or signal-driven readouts, these detection platforms minimize the dependence on complex equipment and advanced laboratory infrastructure, and are therefore recognized as efficient tools for use in decentralized diagnostic systems and resource-limited environments (44–47).

### 4.3 Rolling circle amplification (RCA)

RCA is one of the most advanced DNA amplification technologies at constant temperature, specifically designed for highly sensitive detection of biomolecules. In this technique, a dedicated probe, called a “padlock probe,” is formed into a closed loop after precise recognition of the target sequence and used as the amplification template (48–50). The powerful Φ29 enzyme, known for its precision and exceptional ability to translocate strands, moves continuously on this circular template, producing very long strands of single-stranded DNA containing thousands of repeat units. This process is carried out at a mild temperature of 30 to 37 degrees Celsius and does not require thermocyclers. The RCA reaction in the basal state increases linearly with time, but it can be converted to pseudoexponential amplification using secondary primers or amplification systems (49–51).

RCA is particularly valuable for single-molecule detection, spatial imaging, biosensors, microfluidic systems, and CRISPR-based ultrasensitive diagnostics. The technology allows researchers to detect even minute amounts of DNA or RNA with high accuracy (50, 52). The most important advantages of this technique include the very high fidelity of the Φ29 enzyme, flexible probe design, and the ability to amplify signals at the single-molecule level. However, the need for a probe ligation and cyclization step, as well as the slower speed compared to techniques such as LAMP and RPA, are limitations unless super-branched versions or multi-step amplification systems are used (50). Amplified products in the RCA method are generally detected through fluorescence-based platforms or probe-based systems; approaches that, by increasing diagnostic sensitivity, enable precise tracking of amplification signals and provide a suitable platform for the development of advanced applications in the field of biosensing and molecular diagnostics (36, 53, 54).

### 4.4 Nucleic acid sequence-based amplification (NASBA)

NASBA is an advanced isothermal amplification technology specifically designed for the detection and amplification of RNA molecules. Unlike conventional PCR-based techniques that require thermal cycling, NASBA is performed at a constant temperature of approximately 41 °C, making it ideal for portable systems and rapid point-of-care diagnostics. In this technique, three key enzymes work in concert: reverse transcriptase, which converts the target RNA into complementary DNA; RNase H, which removes the template RNA strand; and T7 polymerase, which produces large amounts of amplified RNA. First, a primer containing the T7 promoter sequence binds to the target RNA, initiating cDNA synthesis. After the RNA strand is degraded by RNase H, the second primer extends into a double-stranded DNA molecule with an active T7 promoter. T7 polymerase then continuously produces antisense RNA, which re-enters the reaction cycle and exponentially amplifies the RNA signal (55).

NASBA has been widely used in the detection of RNA viruses, foodborne pathogens, and RNA biomarkers due to its specific design for RNA. In recent years (2021–2025), several studies have been conducted focusing on increasing the sensitivity of this technique, including the development of nicking-assisted NASBA systems and the integration of this technology into point-of-care diagnostic platforms, which allow for rapid and accurate detection in clinical and field settings (21, 56, 57). The most important advantages of NASBA are its specificity for RNA, the ability to perform the reaction at a constant, low temperature, and the production of high volumes of RNA amplicons, which make them very easy to detect. However, this technique has lower amplification efficiency than some DNA-based technologies, and its success depends on precise adjustment of the enzyme ratio and activity, so any imbalance in the enzyme system can affect reaction performance (56). In NASBA technology, the detection of amplified products is mainly performed through fluorescence-based systems, especially in the form of real-time platforms that provide continuous monitoring of amplification kinetics. This strategy plays an important role in improving the efficiency of molecular diagnostic tools by increasing the sensitivity and accuracy of RNA target detection (36, 53, 54).

### 4.5 Multiple displacement amplification (MDA)

MDA is an isothermal whole-genome amplification technique performed at about 30 °C and based on the activity of the Φ29 DNA polymerase. Due to its very high processivity, strong ability to displace DNA strands, and 3′ → 5′ exonuclease error-correction activity, this enzyme can replicate DNA with high accuracy and produce long fragments. This technique uses random six-stranded primers (random hexamers) that bind to different regions of the genome and initiate DNA synthesis. During the reaction, the Φ29 enzyme continuously synthesizes new strands while simultaneously displacing the previous strands, leading to the formation of branched DNA structures and the production of large amounts of high-molecular-weight DNA (58). One of the outstanding features of the MDA technique is its ability to produce long DNA products with relatively uniform genome-wide coverage. This feature makes MDA a valuable tool for samples with very low DNA amounts, such as in single-cell genomics and metagenomics studies. However, because of its untargeted nature, it is not usually used as the main technique for identifying a specific pathogen (59). In practice, isothermal amplification techniques such as MDA are widely used as pre-amplification strategies to increase the sensitivity of downstream diagnostic techniques for samples with low NA levels. For example, MDA has been used prior to next-generation sequencing (NGS) to improve genomic coverage and analytical sensitivity in low-abundance microbial and viral samples, ultimately leading to more reliable pathogen identification. In addition, isothermal techniques such as Loop-mediated isothermal amplification (LAMP) and recombinase-aided amplification (RAA) are often integrated with lateral flow assays (LFAs) or CRISPR-based diagnostic systems (60). In these hybrid systems, the amplification step increases the concentration of the target NA, significantly improving the detection limit, with sensitivity reaching a few copies per reaction in some cases. Taken together, these integrated approaches not only increase sensitivity and specificity but also allow the development of rapid, portable, and field-ready diagnostic techniques with minimal sample preparation. These features make these techniques highly suitable for veterinary and environmental applications, where rapid and sensitive detection of low-abundance pathogens is of paramount importance (60). MDA is widely used in single-cell genomics, metagenomics, and as a pre-amplification step for long-read sequencing platforms. Recent studies in 2023 and 2024 have continued to confirm the superior performance of the Φ29 enzyme, but have also highlighted the formation of chimeric structures and the need for careful protocol optimization to reduce amplification artifacts (61, 62).

From a practical perspective, the MDA technique offers advantages such as very high accuracy, the ability to amplify DNA from very limited amounts, the production of long fragments, and relatively uniform genome coverage. However, if reaction conditions are not carefully controlled, there is a risk of generating chimeric products and amplification bias in some regions of the genome. Therefore, optimizing reaction conditions, appropriately designing primers, and adhering to contamination control principles are essential for the proper implementation of this technique (61). Identification of amplified products in MDA is generally performed through advanced molecular approaches such as fluorescence-based assays or sequencing-based technologies; the selection of the appropriate method depends on the application goals and analytical requirements of the study (60).

### 4.6 Helicase-dependent amplification (HDA)

HDA is an isothermal technique for DNA replication that mimics the natural mechanism of DNA replication in cells. In this technique, the enzyme helicase continuously unwinds the double-stranded DNA, converting it into single strands. Primers then bind these single strands, and DNA polymerase continues making new strands at a constant temperature. This coordinated cooperation among the helicase, single-stranded DNA-binding proteins (SSBs), and the polymerase allows DNA replication to occur without thermal denaturation and at a single constant temperature. Depending on the type of enzymes used, the reaction is usually carried out at temperatures between 37 and 65 °C (27, 28, 63).

Since HDA does not require complex instrumentation and thermal cycling, it is a very suitable option for portable diagnostic systems, low-power devices, and microfluidic chip-based platforms. The mechanism of this technique is very similar to PCR, except that it is performed at a constant temperature. For this reason, HDA has found wide applications in the field of point-of-care diagnostic tests. Recent scientific reviews have also comprehensively examined the different families of helicase enzymes and how they are adapted for molecular diagnostic applications (27, 28). The HDA technique requires only mild heating and can be easily implemented in small systems-on-a-chip or battery-powered devices (64). Compared with common isothermal techniques such as LAMP and RPA, the technique is more similar in its mechanism of action to natural DNA replication. However, its replication rate is usually slower, and it relies on a multicomponent enzyme complex. Also, sequences with high GC content or complex secondary structures can reduce reaction efficiency; therefore, careful optimization of the helicase-polymerase enzyme pair is essential for the accurate identification of veterinary pathogens (28). Compared to other isothermal amplification techniques, helicase-dependent amplification (HDA) typically requires a longer reaction time. Recombinase-based techniques such as RPA often perform amplification in 15–30 min, while LAMP reactions are typically completed in 30–60 min (24, 65). In contrast, HDA typically requires approximately 60–120 min to achieve a similar level of amplification. However, this time difference is not necessarily considered a major limitation in practical applications. In many clinical and field settings where simplicity, portability, and minimal equipment requirements are a priority, the relative increase in reaction time is acceptable. Furthermore, although techniques such as LAMP have high performance in terms of speed and sensitivity, the stability of the amplification mechanism in HDA and its similarity to PCR could lead to greater reliability and better compatibility with portable diagnostic platforms, which enhances its application in point-of-care diagnostic systems (28, 65). HDA amplification products are typically detected using fluorescence-based or probe-driven systems, which are conceptually similar to those used in PCR diagnostics. These approaches provide reliable and sensitive readouts, while also supporting integration into portable platforms, making them well-suited for use in field and point-of-care settings (36, 53).

### 4.7 Cross-priming amplification (CPA)

CPA is an isothermal NA amplification technique performed at approximately 60–65 °C. The technique uses a set of specifically designed primers, at least one of which is a cross-primer. The 5′ end of this primer contains a sequence that is not directly complementary to the target region but is incorporated into the final product during amplification, allowing for a self-amplifying, exponential amplification cycle (66). The CPA reaction uses the Bst DNA polymerase enzyme with strand-displacement capability, which enables continuous DNA amplification without the need for PCR thermal cycling (67, 68). Thus, the reaction can be carried out using only simple thermal equipment such as a bain-marie or dry heater. The products of the CPA reaction can be identified using simple, rapid techniques, such as colorimetric detection and lateral flow strips. These detection approaches support deployment in non-laboratory settings by enabling rapid, often visual, interpretation of results. By minimizing reliance on advanced instrumentation, they play a key role in expanding decentralized diagnostic capabilities (44–47). CPA has been used to detect a wide range of human and animal pathogens, including *Mycobacterium tuberculosis* and respiratory pathogens, from clinical samples. Recent clinical studies indicate that this technique is increasingly being considered for Point-of-Care Testing (POCT) systems, particularly in the veterinary and food safety fields (67–69). The main advantages of CPA include minimal equipment requirements, the ability to perform the reaction with simple heat sources, the ability to visually detect results, and good tolerance to complex sample matrices. These features make CPA a suitable option for field applications, laboratories with limited facilities, and rapid diagnostic systems. In contrast, compared to more established isothermal techniques such as LAMP and RPA, the CPA technique has not yet been fully standardized. The first design can vary depending on the target sequence and often requires careful experimental optimization, which can limit the uniform and widespread implementation of this technique (67, 68).

### 4.8 Strand-exchange amplification (SEA)

SEA, also known as denaturation-bubble-mediated strand exchange amplification, is a novel isothermal nucleic acid amplification technique that relies on the natural, transient opening and closing of the two DNA strands at moderate temperatures. Under these conditions, the DNA molecule temporarily undergoes short-term openings called denaturation bubbles, which allow primers to bind. After the primers anneal, a strand-displacing polymerase is activated and initiates strand exchange by synthesizing a new strand. This cycle continues at a constant temperature (often ~60 °C), resulting in rapid amplification of the target sequence (70). Unlike techniques such as RPA and HDA that require auxiliary enzymes such as recombinase or helicase, SEA can be performed with only one primer pair and one polymerase. This feature simplifies reaction formulation, reduces procedural complexity, and makes it easier to use across different laboratory settings. In addition, more advanced versions of this technology, such as Accelerated SEA (ASEA), have been developed that significantly increase the speed of detection and allow for ultra-rapid identification of pathogens (70, 71). Detection of amplified products in SEA is usually performed via simple colorimetric or fluorescence detection methods, which allow for rapid and visual readout of results. These features make SEA an efficient tool for field diagnostics and in-situ diagnostic systems, without the need for complex laboratory equipment (33, 53). SEA, as an emerging platform in the field of molecular diagnostics, has attracted widespread attention in the field of rapid, portable, and point-of-care testing. Recent studies have shown that the speed and specificity of this technique can be significantly improved by optimizing primer design, and even visual and colorimetric versions of it can be developed for rapid screening (70). This technique has been used to identify foodborne pathogens such as *Staphylococcus aureus*, forest pathogens, and animal pathogens, demonstrating the high potential of SEA in veterinary diagnostics, food safety, and environmental monitoring (72). The requirement for a minimal number of primers, simple implementation, and complete reaction under isothermal conditions makes SEA a very suitable option for portable diagnostics, field testing, and low-resource environments. However, as SEA is a relatively new technique, there are currently limited commercial kits available for it, and it has not yet undergone as extensive clinical-scale validation studies as techniques such as LAMP and RPA (70). A comparative review of the principal isothermal amplification technologies in veterinary diagnostics, along with a review of the performance characteristics, advantages, and limitations of each, is presented in Table 1.

**Table 1:** Comparison of major isothermal amplification technologies used in veterinary diagnostics.

Technique	Target NA	Operating temperature (°C)	Time to result	Major advantages	Key limitations	References
Loop-mediated isothermal amplification (LAMP)	DNA/RNA (RT-LAMP)	60–65	30–45 min	High sensitivity, visual detection, tolerant to inhibitors	Complex primer design, contamination risk	(18, 38)
Recombinase polymerase amplification (RPA)	DNA/RNA (RT-RPA)	37–42	10–20 min	Very rapid, low temperature, field-friendly	High enzyme cost, primer optimization	(39–43)
Rolling circle amplification (RCA)	DNA/RNA	30–37	60–120 min	High fidelity, single-molecule sensitivity	Slow, ligation step required	(48–52)
Nucleic acid sequence-based amplification (NASBA)	RNA only	~41	60–90 min	RNA-specific, high signal output	Enzyme balance critical	(21, 55–57)
Multiple displacement amplification (MDA)	DNA	~30	2–4 h	Whole-genome amplification, high accuracy	Not target-specific	(58, 59, 61, 62)
Helicase dependent amplification (HDA)	DNA	37–65	60–90 min	PCR-like mechanism, simple heating	Slower, multi-enzyme complexity	(27, 28, 63, 64)
Cross-priming amplification (CPA)	DNA	60–65	60–90 min	Simple equipment, visual readout	Lower sensitivity	(66–69)
Strand exchange amplification (SEA)	DNA	~60	15–30 min	Minimal enzymes, rapid	Limited validation	(70–72)

## 5 Recent technological advances

### 5.1 Primer and polymerase engineering

In recent years, isothermal nucleic acid amplification technologies have undergone significant advances, with the main focus on improving primer design and polymerase engineering. The goal of these efforts is to increase the efficiency and accuracy of testing for complex veterinary samples, such as food animal tissues, meat products, and pet samples, which have always been challenging due to the presence of inhibitors and variable DNA and RNA quality. Continuous improvements in primer design and polymerase engineering have dramatically increased the specificity and accuracy of isothermal amplification assays. In modern approaches, primer design is largely based on computational tools such as Primer3 and Primer-BLAST, which prevent unwanted amplification and nonspecific interactions by selecting targeted sequences (73, 74). In recent years, the integration of machine learning-based methods has also revolutionized this field; these algorithms have made primer design a faster, more accurate, and data-driven process by predicting amplification efficiency and reducing nonspecific noise. In addition, the use of high-throughput computational pipelines has enabled extensive screening of potential primers, helping to increase the stability and reproducibility of results. On the other hand, the development of engineered polymerases with strand-displacing capabilities, especially optimized versions of Bst and Φ29, has led to improved thermal stability, increased amplification efficiency, and better tolerance to inhibitors present in complex matrices. Collectively, these advances have significantly improved the performance, accuracy, and applicability of isothermal amplification systems under real-world conditions (75–77). For this reason, isothermal amplification techniques have become reliable and efficient tools for rapid disease diagnosis in the veterinary and food industries.

Today, with advanced primer design algorithms and clever structural modifications such as loop primers, locked nucleic acid (LNA) bases, and hairpin stabilizers, the time required for LAMP and RPA reactions has been significantly reduced. At the same time, these improvements have led to a decrease in the rate of non-specific amplifications and an increase in test accuracy. This is particularly important in processed veterinary samples, as it allows for faster, more accurate, and more reliable results (15).

In field samples such as meat, feces, and blood, naturally occurring compounds, including hemoglobin, lipids, and salts, can inhibit polymerase activity and reduce the efficiency of molecular reactions. To overcome this challenge, researchers have developed a new generation of polymerases. The concept of “new generation polymerases” refers to a group of DNA polymerase enzymes that have been developed through protein engineering or functional optimization to significantly increase the efficiency of isothermal amplification methods. Unlike conventional polymerases used in PCR, which require thermal cycling and have limited strand displacement, these enzymes are capable of performing the amplification process continuously at a constant temperature (78). Modified polymerases such as Bst and Φ29 are among the most important examples, which increase the accuracy and efficiency of amplification by having high processibility, strong strand displacement activity, and, in some cases, proofreading properties. Recent advances in enzyme engineering have also led to improved thermal stability, resistance to inhibitors, and increased amplification efficiency under complex biological conditions, such that even very small amounts of the target nucleic acid can be detected (78). Also, the superiority of these enzymes in strand displacement over classical polymerases has enabled rapid, efficient, and reliable amplification in isothermal systems. Ultimately, the combination of these features has made new-generation polymerases key tools for the development of rapid, sensitive, and field-based diagnostic methods and using targeted mutagenesis and directed evolution techniques that exhibit higher stability and performance in harsh and complex biological environments (78). These advances allow for direct diagnostic tests on animal tissues without the need for nucleic acid extraction, paving the way for the development of rapid, low-cost, and reliable techniques for molecular diagnostics (18).

### 5.2 Integration with CRISPR–Cas systems

CRISPR–Cas systems have attracted attention in recent years as an advanced and emerging platform for nucleic acid recognition, due to their properties such as high specificity, precise programmability, and rapid response. In these systems, Cas enzymes are guided by a guide RNA (RNA) to recognize complementary sequences in DNA or RNA with high accuracy. Cas12 and Cas13, in particular, can be activated and exhibit “collateral cleavage” activities. These activities respond to the non-specific degradation of labeled reporter molecules in the environment and can ultimately be measured, usually as fluorescence or color dyes, produced. This unique feature allows for very low detection limits and high detection, even when the target is very small. To improve analytical performance, CRISPR-based systems are increasingly being integrated with isothermal amplification techniques such as LAMP and RPA. These features not only increase the synthesis but also significantly reduce the response time, allowing the assays to be performed under simple conditions and without the need for expensive instrumentation. Furthermore, these technologies are adaptable to a variety of signal readout formats, including fluorescence-based systems and lateral flow assays, which are applicable in both laboratory and field settings. Given these features, CRISPR–C systems are presented as a breakthrough in the development of rapid, accurate, and portable diagnostic tools and have high potential for point-of-care diagnosis, disease monitoring, livestock applications, and identification of pathogens in the environment (79–81). The combination of isothermal amplification techniques with CRISPR–Cas12/Cas13 technology has enabled the development of highly sensitive and accurate diagnostic systems useful for detecting veterinary pathogens.

The DNA-targeting Cas12a and RNA-recognizing Cas13a/b enzymes, with the help of programmed guide RNAs, specifically bind to the pathogen’s genetic sequences. Once activated, these enzymes cleave the labeled reporter probes, and the detection result is visible as a fluorescence signal or a strip test (82, 83).

Collateral Cleavage is a natural mechanism for signal amplification that dramatically increases detection power without additional complex steps, enabling the detection of very small amounts of target down to attomolar levels. This feature significantly increases the sensitivity of the diagnostic techniques above that of the original isothermal reactions. In recent years, the combination of isothermal amplification techniques, such as LAMP and RPA, with Cas12 and Cas13 systems has been developed and successfully used for rapid, accurate detection of zoonotic bacteria and viruses. These technologies have provided a new path for ultrasensitive molecular diagnostics that can be used in field conditions (84, 85).

Recent studies have shown that CRISPR-Cas-enhanced LAMP assays can detect Brucella species directly from animal serum samples in less than 40 min (84). Cas13-based systems have also been successfully developed for the detection of RNA viruses in livestock and poultry (85). Combining the simplicity and speed of isothermal amplification with the exceptional accuracy of CRISPR single-nucleotide detection, these integrated techniques provide powerful, practical tools for monitoring pathogens in food-producing and companion animals.

### 5.3 Microfluidic and portable platforms

Microfluidic and portable platforms are a new generation of point-of-care detection systems that utilize lab-on-a-chip technology to integrate NA extraction, isothermal amplification, and CRISPR-based detection in a single cartridge. These systems require very small sample volumes (≤10 μL) and utilize on-chip temperature control, enabling rapid and accurate molecular testing in portable, battery-powered devices (86–88).

The multiplexing capability of modern microfluidic chips allows for the simultaneous detection of multiple veterinary pathogens using isothermal LAMP or RPA reactions. Simultaneous detection of pathogens with fluorescent output readable by a mobile phone has been successfully demonstrated, making this technique a rapid and practical tool for field diagnosis (87, 89).

The use of closed cartridge systems significantly reduces the risk of cross-contamination and false positives compared to open-tube techniques, which is particularly important in environments with high biocontamination, such as livestock farms and slaughterhouses (88). Furthermore, the combination of these systems with lateral flow strips and mobile phone-based analysis allows for semi-quantitative results that can be interpreted by non-specialist users (88).

## 6 Applications for food and companion animal diagnostics

### 6.1 Bacterial detection

LAMP has been widely applied for the detection of *Salmonella* and *Escherichia coli* across diverse food-animal clinical sample types, including chicken and cattle feces, bird viscera, and chicken carcass rinses (90–94). LAMP-based *Salmonella* assays have targeted conserved and serotype-associated genes such as *icIR*, *invA*, *safA* (serotype Enteritidis), *yeeA* (serotype Heidelberg), and *bcfD*, generally demonstrating high analytical specificity (90, 91, 93, 94). However, sensitivity varied by sample matrix, with one LAMP-only approach achieving less than 90% sensitivity in chicken fecal samples (91). In contrast, integration of LAMP with CRISPR-Cas12b detection significantly improved both sensitivity and specificity for *Salmonella* detection in chicken feces, highlighting the value of signal amplification or secondary detection strategies for complex matrices (92). LAMP assays have also been extensively evaluated for *E. coli* detection by targeting avian pathogenic *E. coli* virulence genes (*sitA*, *ompT*, and *traT*), Shiga toxin–producing *E. coli* genes (*stx1* and *stx2*), and conserved markers such as *phoA* and *lamB* (95–97). Across spiked chicken liver and heart tissues, chicken feces, and cattle feces, these assays consistently demonstrated high sensitivity and specificity relative to conventional PCR (95, 97). However, performance limitations were observed in certain applications, as a LAMP–lateral flow immunoassay used for mastitis-associated milk samples produced both false-positive and false-negative results, underscoring the influence of matrix complexity and downstream detection format on assay reliability (96).

RPA–based approaches have emerged as rapid and highly sensitive alternatives to conventional PCR for food-animal pathogen detection. Across multiple sample matrices, including chicken anal swab and tissue, RPA platforms consistently demonstrate high sensitivity and specificity for *Salmonella pullorum* and *Salmonella enteritidis* RPA primer design targeting *Salmonella* species specific genes for molecular markers (*Sdf I*) and virulence (*traJ*) enables robust detection when combined with lateral flow dipstick readouts (17, 98). These findings highlight the potential of RPA-based technologies for rapid, on-site animal testing, although broader validation across diverse matrices remains necessary for routine implementation.

MDA has been successfully applied to bacterial pathogen detection when integrated with complementary technologies that enhance selectivity and downstream analysis. When coupled with immunomagnetic separation, MDA has demonstrated high sensitivity for *Salmonella enterica* in poultry litter samples, exceeding that of conventional culture and PCR-based techniques (99). Beyond LAMP-, RPA-, and MDA-based platforms, additional isothermal amplification strategies including CPA and SEA have been evaluated for *Salmonella* detection in food-animal samples. A CPA-based assay and a SEA-based assay have demonstrated high specificity for *Salmonella* detection from tissue and feces samples from animals suspected of *Salmonella* infections (chickens, ducks, geese, swine) as well as from chickens that were experimentally infected (100, 101). Collectively, these findings support the adaptability of isothermal amplification platforms for bacterial pathogen detection in food animals.

### 6.2 Viral detection

LAMP–based assays have been extensively evaluated for the detection of high-consequence viral pathogens affecting animal populations, demonstrating strong potential for rapid field and food-chain surveillance. For avian influenza virus (AIV), LAMP and RT-LAMP assays have shown high analytical sensitivity and specificity in chicken field samples and spiked nasal swabs, with performance comparable to standard real-time RT-PCR and no observed cross-reactivity with non-target avian or bovine pathogens (102–104). Similarly, LAMP-based detection of Newcastle disease virus (NDV) in inoculated egg samples exhibited equivalent sensitivity and specificity to nested PCR techniques, supporting its reliability for avian disease monitoring (105). Beyond avian pathogens, LAMP assays combined with lateral flow device readouts have been applied to African swine fever virus (ASFV) detection, where enhanced sensitivity was observed relative to real-time PCR, ELISA, and LAMP without lateral flow integration (106). Collectively, these studies highlight the versatility of LAMP platforms for rapid, sensitive detection of zoonotic and livestock-associated viruses, particularly when paired with simple visual readouts suitable for point-of-need and field-based applications.

RPA–based assays incorporating reverse transcription have been widely evaluated for the rapid detection of viral pathogens affecting livestock and poultry. Reverse transcription RPA (RT-RPA) assays have demonstrated high sensitivity and specificity for the detection of multiple avian influenza virus (AIV) subtypes in avian clinical samples and chicken embryos, with performance comparable to conventional RT-PCR (107–109). RT-RPA platforms have also been adapted for Newcastle disease virus (NDV) detection, often coupled with lateral flow dipstick readouts to enable rapid visual interpretation (110, 111). In swine health applications, colorimetric RPA assays employing SYBR Green I dye have been used for African swine fever virus (ASFV) detection in pig samples, exhibiting sensitivities comparable to PCR- and qPCR-based techniques (112, 113). Additional RPA formats incorporating fluorometric detection and single-tube reaction systems have further reduced assay complexity and turnaround time, with some approaches producing results in under 10 min (114). Collectively, these studies underscore the utility of RPA as a rapid, flexible diagnostic platform for high-consequence viral pathogens in both laboratory and field settings.

RCA–based approaches have also been adapted for the detection of high-consequence viral pathogens in animal health surveillance. RCA assays targeting avian influenza virus (AIV) subtype H9N2 have demonstrated high subtype specificity in chicken serum samples, with no cross-reactivity observed against other influenza subtypes such as H1N1 and H3N2 (115). More advanced RCA strategies employing padlock probe cocktails have enabled multiplex detection of avian RNA viruses, including both AIV and Newcastle disease virus (NDV), through simultaneous target enrichment and amplification within microfluidic platforms (116). In swine diagnostics, saltatory RCA coupled with SYBR Green–based visualization has been applied to African swine fever virus (ASFV) detection in clinical samples, achieving complete specificity and higher analytical sensitivity than conventional real-time PCR (117). Together, these findings highlight the adaptability of RCA-based technologies for subtype-specific, multiplex, and highly sensitive viral detection in complex animal-derived samples.

NASBA has been evaluated for the detection of multiple avian influenza virus (AIV) strains across diverse avian sample types, including chicken egg embryos, blood, and anal swabs (118–120). Coupling NASBA with electrochemiluminescent detection has been shown to enhance assay robustness by enabling specific amplification in the presence of field-derived inhibitors and contaminants (118). For African swine fever virus (ASFV), multiple amplification strategies beyond conventional isothermal techniques have been explored to support both detection and genomic characterization. MDA has enabled successful whole-gene amplification and sequencing of ASFV from infected pig blood and serum samples, facilitating downstream genomic analysis (121). In parallel, a fluorescence-based single-tube CPA assay demonstrated sensitivity and specificity equivalent to real-time PCR for ASFV detection in swine blood and serum (122). More recently, graphene oxide–based accelerated SEA assay has been applied to ASFV detection in pig nasal swabs, where the incorporation of graphene oxide reduced nonspecific amplification by suppressing primer dimer formation (123). Collectively, these approaches illustrate the expanding diversity of amplification technologies being leveraged to improve sensitivity, specificity, and robustness for viral pathogen detection in animal health surveillance.

### 6.3 Parasitic detection

LAMP has been increasingly applied for the detection of parasitic pathogens, including *Toxoplasma gondii* and *Cryptosporidium* species, offering a rapid, sensitive, and specific alternative to conventional morphological, immunological, and PCR-based approaches. LAMP detection has also been applied to fecal sample detection from animals, including pigs, cattle, sheep, and horses (124–127). LAMP techniques for *Cryptosporidium* detection have incorporated rapid, on-site visualization techniques by using fluorescent detection reagents or measurements of turbidity from the precipitation of magnesium pyrophosphate (124, 126). Multi-target LAMP platforms integrated with microfluidic chips have allowed for simultaneous detection of *Toxoplasma* and *Cryptosporidium* across multiple samples, achieving reproducible results and sensitivity and specificity comparable to conventional PCR (125). Beyond food-animal applications, LAMP has demonstrated utility in companion-animal diagnostics, detecting *T. gondii* in cat blood, cat feces, and dog blood as well as in pet bird brain tissue (128–130). Collectively, these studies highlight the versatility of LAMP for parasitic pathogen detection across diverse matrices and sample types, consistently outperforming conventional PCR in sensitivity while maintaining high specificity.

RPA and CPA have also been applied to the detection of parasitic pathogens, demonstrating high sensitivity and specificity across animal-derived samples. RPA assays combined with lateral flow strips have enabled specific detection of *Cryptosporidium* species in cattle feces without cross-reactivity with other enteric pathogens (131, 132). For *Toxoplasma gondii*, novel approaches integrating RPA with CRISPR/Cas9 or CRISPR/Cas12a–based lateral flow assays and digital visualization instruments have effectively detected the parasite in blood samples from stray dogs and cats and from cat clinical fecal samples (80, 133). Similarly, CPA-based assays coupled with lateral flow detection have shown high sensitivity and specificity for *T. gondii* in cat feces (134). These studies highlight the adaptability of isothermal amplification platforms, particularly when paired with lateral CRISPR-based technologies and lateral flow detection, to achieve rapid, reliable, and field-deployable diagnostics for parasitic infections in both livestock and companion animals. Table 2 shows selected applications of isothermal amplification technologies for the identification of bacterial, viral, and parasitic agents in veterinary diagnostics and food-producing animals.

**Table 2:** Applications of isothermal amplification techniques in veterinary and food-animal diagnostics.

Pathogen type	Pathogen examples	Amplification technique(s)	Sample matrix	Detection format	References
Bacterial	*Salmonella*, *E. coli. Brucella* spp.	LAMP, RPA, CPA, SEA, LAMP-CRISPR	Feces, meat, milk, Serum	Colorimetric, LFA, fluorescence, CRISPR-Cas fluorescence	(17, 91–101, 103)
Viral	ASFV, AIV, NDV	RT-LAMP, RT-RPA, RCA, MDA	Serum, swabs, tissues, Blood	LFA, fluorescence, Sequencing	(17, 102, 104–110, 112–123, 127)
Parasitic	*Toxoplasma gondii*, *Cryptosporidium*	LAMP, RPA, CPA	Feces, blood	Fluorescence, LFA	(60, 80, 124–126, 129, 130, 133, 134)

## 7 Visualization and readout techniques

As fundamental components of isothermal amplification systems, visualization and readout strategies play a crucial role in the transferability of these technologies from laboratory settings to field and point-of-care applications. Although the interpretation of results is technique-specific and is integrated into the relevant subsections in Section II, it is essential to provide a general framework of detection approaches to understand their common principles and practical significance. In this regard, isothermal amplification products are mainly identified through four main approaches: colorimetric detection, fluorescence, lateral flow assays, and turbidimetric assays. Colorimetric methods, using markers sensitive to pH changes or metal ions, allow for direct, instrument-free readouts and are particularly suitable for resource-poor environments and field applications (33–35). In contrast, fluorescence-based systems, using nucleic acid-binding dyes or sequence-specific probes, offer high sensitivity and the ability to monitor reaction dynamics in real time (36, 53, 54, 60). Lateral flow assays, by integrating amplification and detection on paper substrates, produce visual output in the form of visible bands and are widely used in point-of-care diagnostics (44–47, 135, 136). In addition, turbidimetric methods allow semiquantitative or real-time assessment of reaction progress by monitoring turbidity changes resulting from the formation of reaction by-products (137–139). Collectively, these diagnostic approaches play a key role in expanding the application of isothermal techniques in decentralized diagnostic scenarios, including veterinary medicine, food safety, and environmental monitoring (44, 60).

### 7.1 Challenges and limitations of isothermal amplification assays

Despite the numerous advantages presented by isothermal amplification assays, there are also some technical challenges that may limit their use and scalability (22). For instance, RCA is known to be susceptible to mispriming, which arises from poor or non-specific binding between templates and primers. In RCA, target binding is limited to a single interaction between the target and primer, compared to PCR, CPA, and LAMP, where there is primer binding at multiple locations. Additionally, a major limitation of RCA includes the need for a circular DNA template, the use of complex primer designs, and its linear amplification mechanism in the case of linear RCA, resulting in an overall low yield of amplified products (22).

With regards to CPA, a major constraint is the prolonged amplification times and reduced sensitivity when detecting a trace number of targets, which can hinder its effectiveness in early or low-level pathogen detection. In an experimental study, CPA was reported to detect the target bacterium in pure culture with a 100 fg.μL^−1^ detection limit, and in spiked blood samples with a 700 cfu.mL^−1^ detection limit. Considering that the limits are quite high compared with what is commonly reported for other isothermal assays, it suggests that CPA in its standard form shows low sensitivity (22, 140).

HDA significantly suffers from nonspecific primer binding at constant temperatures. The lack of stringent temperature requirements reduces primer annealing specificity, increasing the likelihood of false-positive results (22).

The sensitivity of RPA is highly dependent on primer and probe design; however, standardized design guidelines and robust software tools are grossly lacking. Due to this, extensive screening of several RPA primer and probe sets is warranted in order to determine the optimal combination (141). The situation is complicated when there are attempts to multiplex, as competition among primer sets for recombinase proteins can result in one target preventing the amplification of another (43). Also, large-scale application of RPA is presumed to be constrained by equipment configuration (142).

A major challenge for LAMP is that it is prone to nonspecific amplification, primarily due to primer-dimer formation. The use of multiple primers increases the risk of hybridization of primers, leading to template-independent amplification and false-positive results (38, 143). Also, LAMP assays are particularly susceptible to contamination, owing to multiple pipetting steps and the use of several highly specific primers. As a result, strict aseptic techniques and careful workflow management are necessary to prevent carryover contamination. Additionally, multiplexing remains a significant challenge for LAMP largely due to the complex nature of designing primers for this assay (20, 38).

In a detailed investigation, the robustness of LAMP in the presence of PCR inhibitors has been explored. The effects of seven common PCR inhibitors (bile salts, calcium chloride, hematin, humic acid, immunoglobulin G (IgG), tannic acid, and urea) on LAMP performance have been evaluated. Some inhibitors were shown to delay amplification onset (bile salts, calcium chloride, IgG, and urea), quench amplicon-dye fluorescence (hematin and tannic acid), or reduce total amplification yield (hematin, tannic acid, and humic acid). Interestingly, the inhibitor concentrations that affected LAMP were comparable to or higher than those impacting PCR. Regardless of the negative impacts of these LAMP inhibitors, end-point detection of LAMP amplicons remained largely unaffected, suggesting that LAMP exhibits greater tolerance to inhibitory substances than PCR (10). In addition, isothermal amplification assays such as LAMP inherently generate large volumes of amplicons, which pose a significant contamination risk; when reaction tubes are opened, especially for downstream detection methods like lateral flow dipstick (LFD) readouts. The amplicons can aerosolize and contaminate work surfaces, gloves, pipettes, and the surrounding air, leading to carryover into subsequent reactions and resulting in false-positive outcomes (144). This situation is exacerbated by the need for post-amplification handling in many protocols and the large quantity of amplified DNA produced, making contamination difficult to control. To address these issues, several mitigation strategies have been proposed, including strict sterile pipetting techniques and physical partitioning of LAMP reactions to reduce cross-contamination (145). Alternative closed-tube systems have also been developed to eliminate the need for tube opening, such as LED-based visualization methods demonstrated in Brucella detection (146) and wax-sealed dye systems that prevent exposure of amplified products to the environment (134). Enzymatic approaches, such as the incorporation of uracil-DNA glycosylase (UDG), further help prevent carryover contamination by degrading previously amplified DNA in one-pot reactions without compromising amplification efficiency (147). More advanced molecular strategies include CRISPR/Cas9-based systems designed to selectively eliminate contaminant DNA, thereby enabling contamination-free LAMP workflows (148). Collectively, these approaches highlight both the inherent susceptibility of isothermal amplification assays to contamination and the range of innovations aimed at mitigating this critical limitation.

### 7.2 Future directions

The next generation of isothermal amplification technologies for screening food and companion animal diseases must address existing limitations while leveraging emerging innovations. Primer design remains a critical challenge for techniques such as LAMP and RPA because of the need for multiple primers targeting distinct genomic regions. Future efforts should focus on integrating artificial intelligence (AI) into primer design workflows. AI-driven tools like *LAMPrimers iQ* already demonstrate the potential of machine learning algorithms to predict secondary structures, minimize cross-hybridization, and ensure high specificity across diverse pathogen genomes. These systems incorporate thermodynamic constraints and process long sequences, significantly reducing design time and improving assay reliability for emerging pathogens in veterinary medicine (149). Expanding these capabilities to include real-time predictive modeling, as seen in digital NA amplification platforms such as dLAMP, will further enhance sensitivity and enable automated primer validation pipelines integrated with cloud-based bioinformatics tools for rapid outbreak response (150).

Another critical direction is multiplex diagnostics, which will be essential for managing co-infections common in livestock and companion animals. Advances in microfluidics combined with LAMP have already enabled platforms such as centrifugal microfluidic disks (CMFD) that can simultaneously identify six major swine viruses within an hour (151). Future research should aim to scale these systems for broader pathogen panels and integrate them with portable devices for on-site testing. Similarly, duplex RPA assays for porcine circoviruses and multiplex LAMP techniques coupled with dipstick chromatography for differential detection of *Mycobacterium bovis* and *M. tuberculosis* illustrate the feasibility of rapid, multi-target detection at the farm level (152). Emerging approaches such as multiplex RT-LAMP using quencher/fluorescence oligonucleotides for influenza subtypes (A/H1, A/H3, and B) (135), and optimized LAMP PCR techniques for CSFV genotypes (153), highlight the need for highly specific, multi-pathogen assays that remain robust across diverse sample matrices. Future innovations should also explore CRISPR-Cas integration with isothermal amplification to enhance specificity and multiplexing capabilities.

AI is increasingly transforming LAMP workflows by improving both result interpretation and upstream sample processing. In terms of readout, AI-integrated systems enable automated image acquisition and analysis, significantly reducing subjectivity associated with traditional colorimetric interpretation (154). Beyond simple visual assessment, AI-driven approaches facilitate quantitative analysis of LAMP curves, thereby enhancing accuracy and reproducibility in result interpretation workflows (155). These automated imaging and processing pipelines also minimize human handling, which not only standardizes outputs but reduces contamination risks associated with manual intervention (154). Complementing these advances, smartphone-based RT-LAMP platforms further streamline diagnostics by integrating sample mixing, lysis, amplification, and fluorescence detection into portable systems; for example, handheld devices using 3D-printed cartridges and phone-based imaging can detect SARS-CoV-2 from swab samples in under 40 min without the need for conventional laboratory infrastructure (156). Additionally, machine learning models trained on diverse biological matrices (for instance, swabs, tissues, and poultry feces) can predict optimal lysis conditions, buffer compositions, and enzyme formulations to maximize nucleic acid yield while minimizing the impact of inhibitors. This is exemplified in RT-LAMP systems where pre-heating at 65 °C enables simultaneous lysis and reverse transcription, eliminating the need for separate RNA extraction steps (157). Furthermore, AI-driven models can analyze spectroscopic and imaging data from crude lysates to identify the presence of amplification inhibitors, such as heme or humic substances, and simulate preprocessing parameters, including centrifugation speeds or chelator use, to optimize assay sensitivity. These developments reflect broader trends in AI-enhanced molecular diagnostics, where predictive modeling is used to refine and standardize workflows across diverse and challenging sample types (158).

Finally, Internet of Things (IoT) and cloud-based integration will redefine how diagnostic data is collected and analyzed to enhance policy decision-making. IoT-enabled devices, combined with cloud analytics, can enable the real-time transmission of diagnostic results from portable LAMP platforms or wearable sensors via networks such as LoRaWAN or NB-IoT. These systems will support centralized dashboards for predictive analytics, outbreak prevention, and resource optimization in precision livestock farming. Current prototypes, such as paper-based sensor systems for point-of-care NA amplification tests using Arduino microcontrollers and Wi-Fi connectivity, demonstrate the feasibility of real-time monitoring (159). Cloud integration through smartphone-based optical readouts of LAMP assays simplifies data processing and remote interpretation (160). Future research should build on IoMT-based designs that leverage WebSockets for optimized communication and real-time validation (161), as well as low-cost molecular test platforms using Bluetooth-enabled spectrometers and smartphone apps for rapid pathogen detection, with anonymized data transmission to public health systems (162). These innovations collectively point toward scalable, connected diagnostic ecosystems that enable frequent testing, early intervention, and improved animal health management.

## 8 Conclusion

In the last two decades, isothermal NA amplification techniques have been recognized as one of the most important advances in the field of molecular diagnostics in human and veterinary medicine. By performing the reaction at a constant temperature and eliminating the need for thermal cycling, these techniques have enabled rapid, sensitive, and portable detection of pathogens. For this reason, their application has expanded beyond specialized laboratories to farms, slaughterhouses, and field environments (23, 88). Among these technologies, techniques such as LAMP and RPA have enabled the detection of viruses, bacteria, and parasites in a short time and with minimal equipment. The use of simple outputs, such as color changes, fluorescence signals, or lateral flow bands, makes interpreting results easy, making these tests very useful for decentralized diagnosis and rapid decision-making (18, 25).

In recent years, the combination of isothermal amplification techniques with CRISPR-Cas-based diagnostic systems and microfluidic technologies has significantly increased the accuracy and specificity of detection. This integration not only allows the simultaneous detection of multiple pathogens but also improves the practical efficiency of these techniques by reducing the risk of cross-contamination and reducing reagent consumption (82, 84).

Together, these advances have made isothermal techniques a key component of modern veterinary disease surveillance and diagnosis systems. These technologies play an important role in realizing the “One Health” approach, which addresses the interrelationship between human, animal, and environmental health by enabling early detection, more effective management of disease outbreaks, and improved food safety (85).

Despite these achievements, further progress in this field requires wider standardization of tests, evaluation of their performance under real-world conditions and in the presence of complex samples, and ensuring their cost-effectiveness, especially in resource-poor settings. With the development of more inhibitor-resistant enzymes and the increasing multiplexing power of CRISPR-based and microfluidic systems, isothermal amplification techniques are expected to play a more prominent role in the future of veterinary diagnostics. These field tools enable rapid, evidence-based decision-making for veterinarians, inspectors, and producers, facilitating the timely implementation of control measures such as quarantine, treatment, or product recalls to protect animal and human health and the food supply chain.
